# Which Factors May Contribute to a Successful Outcome in Hemophagocytic Lymphohistiocytosis: A Case Report

**DOI:** 10.7759/cureus.113854

**Published:** 2026-08-03

**Authors:** Adriana Costa, Francisca A Correia, Ana Margarida Fonseca, Vanessa Chaves, Jorge Almeida

**Affiliations:** 1 Internal Medicine, Unidade Local de Saúde São João, Porto, PRT

**Keywords:** breast cancer, chemotherapy-related toxicity, dexamethasone, hscore, secondary hemophagocytic lymphohistiocytosis

## Abstract

Hemophagocytic lymphohistiocytosis (HLH) is a rare and life-threatening hyperinflammatory syndrome characterized by immune dysregulation and excessive cytokine activation. In adults, HLH is most commonly secondary to infections, malignancies, autoimmune diseases, or drug exposure.

We report the case of a 50-year-old woman undergoing adjuvant treatment for human epidermal growth factor receptor 2 (HER2)-positive breast cancer who developed acute hypoxemic respiratory failure, neurological impairment, bicytopenia, marked hyperferritinemia, hypertriglyceridemia, and hepatomegaly. Extensive infectious, autoimmune, and neoplastic investigations were negative. The HScore, a validated diagnostic score for estimating the probability of secondary HLH in adults, was calculated at 211, corresponding to an estimated 93-96% probability of HLH. Following bone marrow examination demonstrating hemophagocytosis, the HScore increased to 246 (>99% probability). Chemotherapy was considered the probable trigger after exclusion of alternative causes. Treatment was initiated according to the HLH-94 protocol with dexamethasone, resulting in prompt clinical and laboratory improvement. The patient achieved full hematologic recovery and resumed oncologic therapy without HLH recurrence.

This case highlights the importance of maintaining a high index of suspicion for HLH, systematic evaluation for underlying triggers, and prompt initiation of immunosuppressive therapy to improve patient outcomes.

## Introduction

Hemophagocytic lymphohistiocytosis (HLH) is a rare, life-threatening hyperinflammatory syndrome characterized by uncontrolled immune activation. It may occur as a primary (familial) disorder caused by inherited defects in lymphocyte-mediated immune regulation or as a secondary condition triggered by infections, autoimmune diseases, malignancies, or medications, including chemotherapy. Although a genetic predisposition may contribute to disease susceptibility, the pathophysiology of secondary HLH remains incompletely understood [1,2].

Although HLH has traditionally been considered a pediatric disorder, approximately 40% of cases occur in adults. Prognosis varies considerably according to patient age and, especially, the underlying trigger. Patients with malignancy-associated HLH have particularly poor outcomes, with substantially lower survival rates than those with other forms of secondary HLH [1-3].

Diagnosing HLH in adults remains particularly challenging because its clinical and laboratory manifestations are nonspecific and frequently overlap with those of the underlying triggering condition. Consequently, delayed recognition is common and may adversely affect prognosis. Although the HLH-2004 diagnostic criteria were originally developed for pediatric patients and have limitations when applied to adults, they remain widely used alongside the HScore in adult clinical practice. In adults, the HScore is a validated diagnostic tool that estimates the probability of secondary HLH using a combination of clinical features, laboratory findings, and bone marrow examination, and is widely used to support diagnostic evaluation in adult patients [4-8].

Early recognition of HLH, prompt identification of the underlying trigger, and timely initiation of immunosuppressive therapy are essential to improve clinical outcomes. This case highlights the importance of maintaining a high index of suspicion for HLH in adults with compatible clinical and laboratory findings and illustrates how systematic exclusion of alternative causes, prompt initiation of immunosuppressive therapy, and withdrawal of the probable precipitating factor were associated with a favorable clinical outcome.

## Case presentation

A 50-year-old woman presented to the emergency department with generalized myalgias and new-onset hypoxemic respiratory failure. She denied fever, cough, chest pain, or other focal symptoms. Initial investigations excluded infection and pulmonary thromboembolism, and she was admitted to the Internal Medicine ward for further evaluation.

Her medical history was significant for ulcerative colitis, diagnosed in 2016 and well controlled with topical mesalazine, and HER2-positive breast cancer diagnosed diagnosed earlier that year. She had undergone right mastectomy with right axillary lymph node dissection, followed by adjuvant chemotherapy with doxorubicin and cyclophosphamide (discontinued after four cycles) and one cycle of trastuzumab. Her medical history also included hypertension, dyslipidemia, overweight, and former smoking.

She reported no relevant family history, recent travel, high-risk exposures, alcohol or illicit drug use, or other epidemiological risk factors for infectious disease.

On the first day of hospitalization, the patient developed altered mental status. Physical examination revealed drowsiness and psychomotor slowing without focal neurological deficits. Brain computed tomography excluded intracranial metastases and acute cerebrovascular events. Laboratory investigations revealed bicytopenia, markedly elevated ferritin and lactate dehydrogenase levels, hypertriglyceridemia, and mild hepatic cytolysis (Table 1).

**Table 1:** Initial laboratory findings at the time of suspicion for hemophagocytic lymphohistiocytosis

Parameter	Patient value	Reference range
Hemoglobin (g/dL)	10.6	12–16
Platelet count (× 10⁹/L)	27	150–459
AST(U/L)	78	10–31
LDH (U/L)	3309	135–225
Ferritin (ng/mL)	12,359	20–250
Triglycerides (mg/dL)	331	<150

Abdominal ultrasonography revealed hepatomegaly without splenomegaly. 

An extensive investigation was undertaken to identify potential secondary triggers (Table 2).

**Table 2:** Investigation performed to identify potential triggers of secondary hemophagocytic lymphohistiocytosis (HLH)

Investigation	Result
Blood cultures	Negative (multiple sets)
Urine culture	Negative
Cerebral fluid analysis	No evidence of central nervous system infection (1 leukocyte/uL, normal protein and glucose)
Cerebral fluid bacterial culture	Negative
Cerebral fluid PCR (HSV-1, HSV-2, Borrelia burgdorferi, Listeria monocytogenes)	Negative
HIV serology	Negative
Hepatitis B virus (HBV) serology	Negative
Hepatitis C virus (HCV) serology	Negative
Syphilis (TPPA)	Non-reactive
Cytomegalovirus (CMV) serology	Negative IgM e IgG
Epstein–Barr virus (EBV) serology	Pattern consistent with past infection (VCA IgG and EBNA IgG positive; IgM negative)
Herpes simplex virus (HSV-1/HSV-2) serology	Past immunity (IgG positive, IgM negative)
Borrelia burgdorferi serology	Negative
Parvovirus B19 serology	Negative
Rickettsia conorii serology	Negative
Whole-body CT	No evidence of metastatic disease or infectious focus
Brain MRI	No abnormal parenchymal or meningeal enhancement
FDG-PET	No evidence of infectious, inflammatory, or neoplastic pathology

Multiple blood and urine cultures were negative, and cerebrospinal fluid analysis excluded central nervous system infection. Serological and molecular testing for infectious agents, including HIV, hepatitis B virus (HBV), hepatitis C virus (HCV), cytomegalovirus (CMV), Epstein-Barr virus (EBV), Herpes Simplex virus (HSV), Borrelia burgdorferi, Parvovirus B19, and Rickettsia conorii, showed no evidence of active infection. Whole-body imaging excluded metastatic disease, while brain magnetic resonance imaging and positron emission tomography revealed no evidence of infectious, inflammatory, or neoplastic pathology.

The coexistence of bicytopenia, marked hyperferritinemia, hypertriglyceridemia, elevated liver enzymes, hepatomegaly, and neurological dysfunction prompted suspicion of HLH. The calculated HScore was 211, corresponding to an estimated 93-96% probability of HLH.

**Figure 1:**
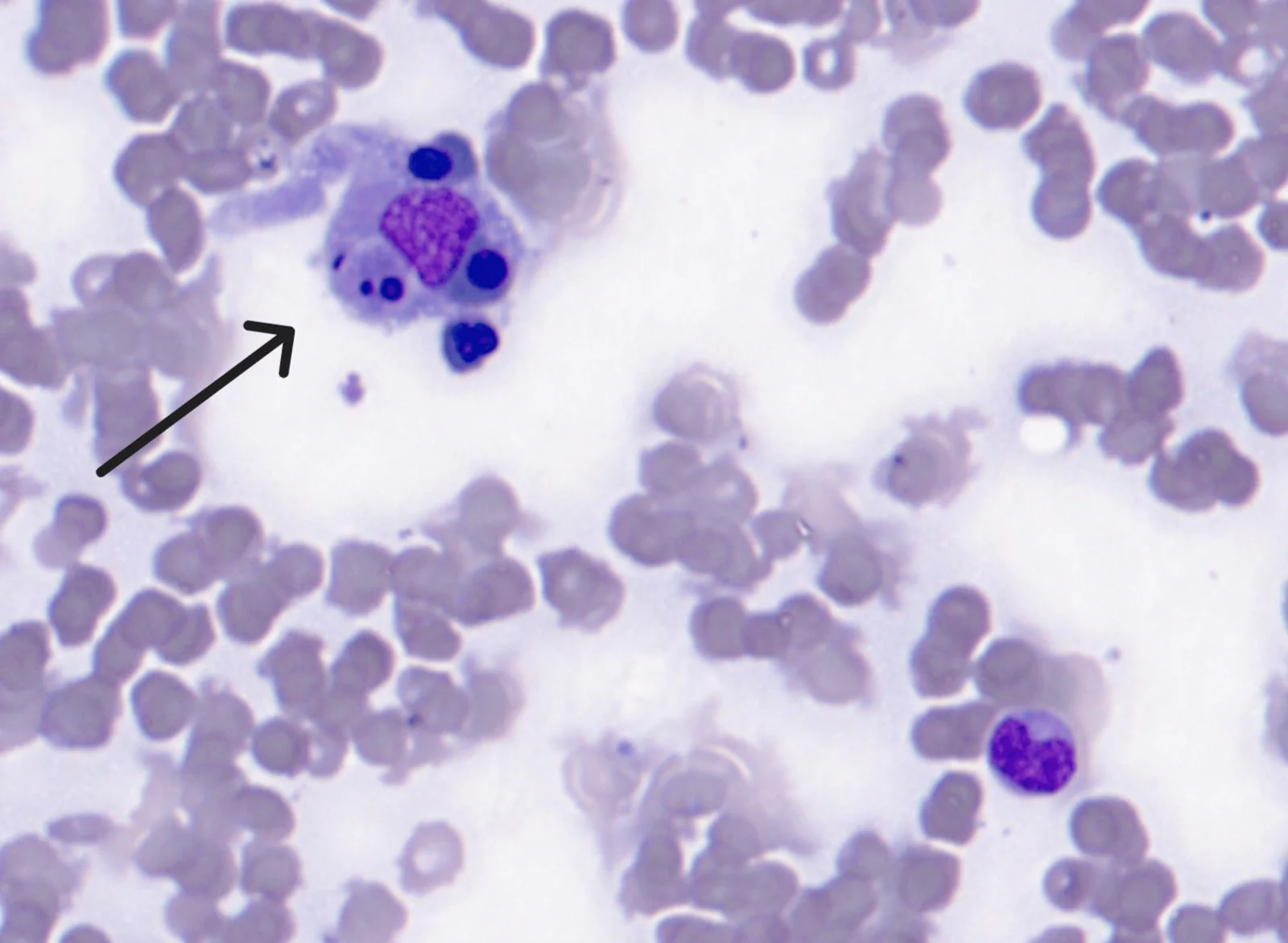
Bone marrow aspirate demonstrating hemophagocytosis The arrow indicates a macrophage exhibiting hemophagocytosis. Wright stain, ×1000 magnification.

Following systematic exclusion of infectious, autoimmune, and neoplastic causes, chemotherapy was considered the most likely trigger for secondary HLH. Bone marrow aspirate demonstrated hemophagocytosis (Figure 1) without evidence of metastatic breast carcinoma.

Incorporation of the bone marrow findings increased the HScore to 246, corresponding to a 99% probability of HLH. NK-cell activity testing was unavailable at our institution, and soluble interleukin-2 receptor (sCD25) levels remained below the diagnostic threshold.

Given the high clinical suspicion of HLH, treatment with dexamethasone (10 mg/m²) was initiated according to an adapted HLH-94-based approach, resulting in progressive clinical and laboratory improvement [3]. She was discharged after 17 days under a corticosteroid tapering regimen. 

The patient was followed up at two weeks and one month after hospitalization. At the one-month follow-up, she was asymptomatic, with marked improvement and near normalization of the laboratory abnormalities (Table 3).

**Table 3:** Evolution of key laboratory parameters throughout the patient's clinical course

Laboratory parameter	At HLH diagnosis	1-month follow-up	Reference range
Hemoglobin (g/dL)	10.6	11.6	12–16
Platelet count (× 10⁹/L)	27	173	150–459
AST (U/L)	78	26	10–31
LDH (U/L)	3309	478	135–225
Ferritin (ng/mL)	12,359	108	20–250
Triglycerides (mg/dL)	331	150	<150

Regarding her oncological treatment, anastrozole was initiated and trastuzumab therapy was resumed one month after discharge. The patient successfully completed corticosteroid tapering without complications, with no evidence of HLH recurrence after four years.

## Discussion

Secondary HLH remains a diagnostic challenge in adults because its clinical manifestations are nonspecific and frequently overlap with those of infection, malignancy, and autoimmune disease.

Although the HLH-2004 criteria were originally developed for pediatric patients, they remain widely applied in adults despite recognized limitations. According to the HLH-2004 protocol, diagnosis is supported when at least five of eight features are present (Table 4) [9-11].

**Table 4:** Hemophagocytic Lymphohistiocytosis-2004 criteria fulfilled (five out of eight)

HLH-2004 diagnostic criteria	Patient findings	Fulfilled
Fever	Yes	✓
Splenomegaly	No	✗
Cytopenias affecting ≥2 cell lines	Hemoglobin 10.6 g/dL; Platelets 27 ×10⁹/L	✓
Hypertriglyceridemia and/or hypofibrinogenemia	Triglycerides 331 mg/dL	✓
Hemophagocytosis	Bone marrow aspirate positive	✓
Ferritin ≥500 ng/mL	12,359 ng/mL	✓
Low/absent NK-cell activity	Not available	Not assessed
Elevated soluble CD25	Below diagnostic cut-off	✗

In our patient, the combination of bicytopenia, hyperferritinemia, hypertriglyceridemia, hepatomegaly, neurological involvement, and elevated liver enzymes prompted early consideration of HLH. The HScore further supported the diagnosis, and bone marrow aspiration demonstrated hemophagocytosis, providing additional evidence in the overall diagnostic assessment (Table 5) [3-6].

**Table 5:** HScore before and after bone marrow aspiration HScore before bone marrow aspirate: 211 (93–96% probability). Total HScore after bone marrow aspirate: 246 (99% probability).

HScore parameter	Patient finding	Score
Known underlying immunosuppression	Yes (active chemotherapy)	18
Temperature	38.5 °C	33
Organomegaly	Hepatomegaly only	23
Cytopenias	Two cell lineages	24
Ferritin	12,359 ng/mL (>6,000)	50
Triglycerides	331 mg/dL (≈3.74 mmol/L)	44
Fibrinogen	637 mg/dL (6.37 g/L)	0
AST	78 U/L (≥30 U/L)	19
Hemophagocytosis on bone marrow aspirate	Absent before aspirate / Present after aspirate	0 / 35

Identifying the underlying trigger is a fundamental component of HLH management, as treatment should address both the hyperinflammatory state and the precipitating condition. An extensive investigation excluded infectious, autoimmune, and neoplastic progression as potential triggers. Given the temporal relationship with treatment and the absence of alternative etiologies, chemotherapy was considered the most likely precipitating factor. Although chemotherapy-associated HLH is uncommon, it has been increasingly recognized in adult patients receiving cytotoxic or targeted therapies [9]. Chemotherapy-associated HLH has been described only in isolated case reports and small case series, most commonly following cytotoxic agents and, less frequently, anti-HER2 therapies. Our case adds to the limited literature by describing successful management with withdrawal of the suspected precipitating agent and corticosteroid therapy, followed by safe resumption of trastuzumab without HLH recurrence.

Prompt discontinuation of the suspected offending agent, together with early initiation of immunosuppressive therapy favored the favorable clinical outcome observed in this patient. Following successful corticosteroid tapering, no recurrence of HLH was observed. After multidisciplinary evaluation, trastuzumab and endocrine therapy with anastrozole were resumed, whereas doxorubicin and cyclophosphamide were not reintroduced because of their presumed association with HLH. The patient remained clinically well, with no evidence of HLH recurrence during follow-up [9,12,13].

The favorable outcome observed in this patient was likely multifactorial. Early recognition enabled prompt initiation of immunosuppressive therapy before irreversible organ damage developed, while systematic exclusion of alternative etiologies supported identification and withdrawal of the probable precipitating agent. Following multidisciplinary discussion, trastuzumab and hormonal therapy were safely resumed, whereas doxorubicin and cyclophosphamide were permanently discontinued because of their presumed association with HLH. No recurrence of HLH was observed during follow-up. Although the optimal timing and choice of anticancer therapy after chemotherapy-associated HLH remain uncertain, management should be individualized through multidisciplinary decision-making, balancing oncological benefit against the risk of HLH recurrence [12,13].

To our knowledge, reports of HLH associated with adjuvant treatment for HER2-positive breast cancer remain scarce, particularly those documenting successful reintroduction of trastuzumab without recurrence of HLH.

## Conclusions

HLH is a rare but life-threatening hyperinflammatory syndrome that requires a high index of suspicion for timely diagnosis. This case illustrates the diagnostic challenges posed by the nonspecific presentation of secondary HLH in adults. It highlights the importance of considering this diagnosis in patients with unexplained cytopenias, marked hyperferritinemia, and systemic inflammation. Systematic exclusion of alternative etiologies was essential to identify chemotherapy as the most likely precipitating factor. Early initiation of appropriate immunosuppressive therapy together with withdrawal of the presumed trigger was associated with a favorable clinical outcome. Greater awareness of this uncommon entity may facilitate earlier diagnosis and improve patient outcomes.
